# *Begoniapseudoedulis*, a new species in Begoniasect.Platycentrum (Begoniaceae) from southern Guangxi of China

**DOI:** 10.3897/phytokeys.182.69074

**Published:** 2021-10-01

**Authors:** Xin-Xin Feng, Yan Xiao*, Zhi-Xian Liu, Ren-Kun Li, Dan Wei, Dai-Ke Tian

**Affiliations:** 1 Dongguan Botanical Garden, Dongguan 523086, China Dongguan Botanical Garden Dongguan China; 2 College of Life Sciences, Hunan Normal University, Changsha, 410081, China Hunan Normal University Changsha China; 3 Shanghai Chenshan Plant Science Research Center, Chinese Academy of Sciences, 3888 Chenhua Road, Songjiang, Shanghai 201602, China Shanghai Chenshan Plant Science Research Center, Chinese Academy of Sciences Shanghai China; 4 Shanghai Key Laboratory for Plant Functional Genomics and Resources, Shanghai Chenshan Botanical Garden, 3888 Chenhua Road, Songjiang, Shanghai 201602, China Shanghai Chenshan Botanical Garden Shanghai China; 5 Enshi Dongsheng Plant Development Co. Ltd., Enshi 445000, China Enshi Dongsheng Plant Development Co. Ltd. Enshi China; 6 Guangdong Academy of Forestry, Guangzhou 510520, China Guangdong Academy of Forestry Guangzhou China

**Keywords:** Molecular evidence, morphology, new taxon, southern China, taxonomy

## Abstract

*Begoniapseudoedulis*, a new species in Begoniasect.Platycentrum (Klotzsch) A.DC. (Begoniaceae) from southern Guangxi of China, is here described and illustrated. It morphologically resembles *B.edulis* H.Lév. and *B.dielsiana* E.Pritz. ex Diels but differs easily by its hairy petioles and inflorescences, and red hispidulous flower tepals, ovary and capsules. The molecular phylogenetic analysis based on ITS supported that the new species was a monophyletic lineage, separating from both *B.dielsiana* and *B.edulis*. Due to its isolated distribution with several small populations, which are possibly disturbed by human activities, the species is considered as “Near Threatened” (NT) according to the IUCN Red List Categories and Criteria.

## Introduction

As a pan-tropically distributed and the sixth largest genus in the angiosperms (Hughes et al. 2015–onwards; Moonlight et al. 2018), *Begonia* L. consists of 2007 known species belonging to 70 sections (Hughes et al. 2015–onwards; Moonlight et al. 2018) . Although showing a very high diversity in the specific level, the classification and phylogeny of this mega genus remain uncertain due to inadequate field surveys, high similarity and variation in morphology, and wild hybridization (Moonlight et al. 2018; Tian et al. 2018a). *Begonia* species are commonly sensitive to the environment, particularly with a low tolerance to both high and low temperature and strong sunlight. In addition, due to their high ornamental and medicinal values, some wild begonias have been commercially over-collected (Tian et al. 2018a). The vulnerability of the restricted distribution and the fact that it is easily disturbed by human activities has resulted in many *Begonia* species becoming endangered (Shui and Chen 2018).

In the past 20 years, the recognized *Begonia* species increased from 80 to 200 in China (Tian et al. 2018b). Over 220 species belonging to 10 sections have been illustrated in China, which is one of the diversity centers of Asian begonias. China plays a more and more significant role in classical taxonomy, phylogeny, utilization, and diversity conservation of *Begonia* (Tian et al. 2018a, 2018b).

In China, the reported *Begonia* taxa are mainly distributed in southeastern Yunnan and southwestern Guangxi. Over 90 species have been described from Guangxi, most of which belong to B.sect.Coelocentrum (Gu et al. 2007; Dong and Liu 2018). We conducted field surveys in several places in southwestern Guangxi on April 1, 2015, November 13, 2016, and in October 2019, respectively and found a putative new *Begonia* species similar to *B.edulis* H.Lév. and *B.dielsiana* E.Pritz. ex Diels in morphology. In October 2020, the type specimens with flowers were collected from Shiwanshan Mountain. Based on further detailed morphological observation and comparison with its allied species and molecular phylogenetic evidence, the species is confirmed as a new one belonging to B.sect.Platycentrum (Klotzsch) A.DC.

## Materials and methods

### Taxonomic observation

The morphological traits were observed and recorded both in the field and from specimens. The photographs were taken during field surveys in southern Guangxi in China. The specimens are deposited at Chenshan Herbarium (**CSH**) of Shanghai Chenshan Botanical Garden and Herbarium of Sun Yat-sen University (**SYS**), respectively.

### DNA sequencing and molecular analysis

The 16 species from sect. Platycentrum and 9 species from other sections of *Begonia* native to mainland China were selected to reconstruct the phylogenetic relationships (Table 1). *B.dregei* Otto & A.Dietr. of sect. Augustia (Klotzsch) A.DC. from South Africa was used as outgroup in the phylogenetic analysis. The methods for DNA extraction, amplification and data analysis were adopted from Tian et al. (2014, 2015). The nuclear ribosomal DNA (nrDNA) internal transcribed spacer (ITS) region was amplified with primers from Chung et al. (2014). The PCR products of ITS were directly sent to Tsingke Biotechnology Co., Ltd. (Shanghai, China) to be sequenced.

**Table 1.** *Begonia* species and populations included in the phylogenetic analysis (Sectional placement follows Moonlight et al. 2018).

Taxon	Origin	Genbank accession no.	Section	Collector, voucher (Herbarium)
*B.acetosella* Craib	Sapa,Vietnam	AF485102	*Platycentrum*	Forrest, L.L.108 (E)
*B.biflora* T.C.Ku	Malipo, Yunnan, China	JF975965	*Coelocentrum*	Shui, Y.M. et al. 20484 (KUN)
*B.chingii* Irmsch.	Napo, Guangxi, China	KP710820	*Reichenheimia*	Tian, D.K., Li, C. TDK785 (CSH)
*B.circumlobata* Hance	Xinyi, Guangdong, China	KP710815	*Platycentrum*	Tian, D.K., Li, X.P. TDK866 (CSH)
*B.circumlobata* Hance	Youxi, Fujian, China	MZ145342	*Platycentrum*	Tian, D.K., et al. TDK2541(CSH)
*B.lipingensis* Irmsch.	Liping, Guizhou, China	MZ145346	*Platycentrum*	Xiao, Y., et al. XY01 (CSH)
*B.dielsiana* E.Pritz. ex Diels	Wulong, Chongqing, China	KP710805	*Platycentrum*	Tian, D.K., Tian, L.Z. TDK2356 (CSH)
*B.dregei* Otto & Dietr	South Africa	AF469126	*Augustia*	Forrest, L.L.241 (E)
*B.edulis* H.Lév.	Bama, Guangxi, China	KP710813	*Platycentrum*	Tian, D.K., Li, C. TDK757 (CSH)
*B.edulis* H.Lév.	Debao, Guangxi, China	MZ145343	*Platycentrum*	Tian, D.K., et al. TDK3111_14(CSH)
*B.edulis* H.Lév.	Jingxi, Guangxi, China	MZ145344	*Platycentrum*	Tian, D.K., et al. TDK3101_19(CSH)
*B.emeiensis* C.M.Hu ex C.Y .Wu & T.C.Ku	Emeishan, Sichuan, China	KP710816	*Platycentrum*	Tian, D.K., Tian, L.Z. TDK2249 (CSH)
*B.fimbristipula* Hance	Fangchenggang, Guangxi, China	KP710826	*Diploclinium*	Li, C. Yang, L.H. TDK2268 (CSH)
*B.grandis* Dryand.	Yongshun, Huhan, China	KP710828	*Diploclinium*	Li, X.P . Li, X.J. LXJ022 (CSH)
*B.handelii* Irmsch,	Fengshan, Guangxi, China	KP710818	*Platycentrum*	Tian, D.K., Li, C. TDK763 (CSH)
*B.huangii* Y.M.Shui & W.H.Chen	Gejiu, Yunnan, China	JF976001	*Coelocentrum*	Shui, Y.M., et al. 40782 (KUN)
*B.jinyunensis* C.-I Peng, Ding & Q. Wang	Jinyunshan, Chongqing, China	MZ145345	*Platycentrum*	Tian, D.K., et al. TDK623 (CSH)
*B.laminariae* Irmsch.	Pingbian, Yunnan, China	KP710814	*Platycentrum*	Tian, D.K., Li, C. TDK1338 (CSH)
*B.longifolia* Blume	Wuming, Guangxi, China	MZ145347	*Platycentrum*	Tian, D.K., et al. TDK3007_8 (CSH)
*B.palmata* D.Don.	Jinxiu, Guangxi, China	MZ145348	*Platycentrum*	Li, C., Yang, L.H. TDK1848_1(CSH)
*B.palmata* D.Don.	Jinxiu, Guangxi, China	MZ145349	*Platycentrum*	Li, C., Yang, L.H. TDK1848_2 (CSH)
*B.palmata* D.Don.	Jinxiu, Guangxi, China	MZ145350	*Platycentrum*	Li, C., Yang, L.H. TDK1848_3 (CSH)
*B.pedatifida* H.Lév.	Tianlin, Guangxi, China	KP710809	*Platycentrum*	Tian, D.K., Tian, L.Z. TDK1924 (CSH)
*B.pedatifida* H.Lév.	Tianlin, Guangxi, China	KP710810	*Platycentrum*	Tian, D.K., Li, C. TDK774 (CSH)
*B.pseudoedulis* D.K.Tian, X.X.Feng & R.K.Li	Fangchenggang, Guangxi, China	MZ145352	*Platycentrum*	Tian, D.K., Tian, L.Z. TDK2428_2 (CSH)
*B.pseudoedulis* D.K.Tian, X.X.Feng & R.K.Li	Fangchenggang, Guangxi, China	MZ145353	*Platycentrum*	Tian, D.K., Tian, L.Z. TDK2428_3 (CSH)
*B.pseudoedulis* D.K.Tian, X.X.Feng & R.K.Li	Fangchenggang, Guangxi, China	MZ145354	*Platycentrum*	Tian, D.K., Tian, L.Z. TDK2428_4 (CSH)
*B.pseudoedulis* D.K.Tian, X.X.Feng & R.K.Li	Fangchenggang, Guangxi, China	MZ145355	*Platycentrum*	Tian, D.K., Tian, L.Z. TDK2423_5 (CSH)
*B.pseudoedulis* D.K.Tian, X.X.Feng & R.K.Li	Fangchenggang, Guangxi, China	MZ145356	*Platycentrum*	Tian, D.K., Tian, L.Z. TDK2423_7 (CSH)
*B.pseudoedulis* D.K.Tian, X.X.Feng & R.K.Li	Wuming, Guangxi, China	MZ145357	*Platycentrum*	Tian, D.K., et al. TDK3008_1 (CSH)
*B.pseudoedulis* D.K.Tian, X.X.Feng & R.K.Li	Wuming, Guangxi, China	MZ145358	*Platycentrum*	Tian, D.K., et al. TDK3008_4 (CSH)
*B.pseudoedulis* D.K.Tian, X.X.Feng & R.K.Li	Wuming, Guangxi, China	MZ145359	*Platycentrum*	Tian, D.K., et al. TDK3008_5 (CSH)
*B.pseudoedulis* D.K.Tian, X.X.Feng & R.K.Li	Shangsi, Guangxi, China	MZ153095	*Platycentrum*	Feng, X.X., et al.FXX201001 (CSH)
*B.pulchrifolia* D.K.Tian & C.H.Li	Meinvfeng, Leshan, Sichuan, China	KP710811	*Platycentrum*	Tian, D.K., et al. TDK2243 (CSH)
*B.ruboides* C.M.Hu ex C.Y.Wu & T.C.Ku	Hekou, Yunnan, China	JF976047	*Diploclinium*	Shui, Y .M. D-38 (KUN)
*B.scorpiuroloba* D.K.Tian & Q.Tian	Fangchenggang, Guangxi, China	MZ145351	*Platycentrum*	Li, C. Yang, L.H. TDK2269 (CSH)
*B.setifolia* Irmsch.	Lvchun, Yunnan, China	KP710827	*Diploclinium*	Tian, D.K., Li, C. TDK1280 (CSH)
*B.silletensis* (A.DC.) C.Clarke	Lincang, Yunnan, China	AF048988	*Platycentrum*	X.J.Y.01012 (KUN)
*B.wenshanensis* C.M.Hu ex C.Y.Wu & T.C.Ku	Kunming Botanic Garden, China	AF048974	*Diploclinium*	X.J.Y.01010 (KUN)
*B.wilsonii* Gagnep.	Nanchuan, Chongqing, China	KP710819	*Diploclinium*	Tian, D.K., Tian, L.Z. TDK2111 (CSH)

The phylogenetic analysis of Bayesian inference (**BI**) was performed in MrBayes v3.2.7 (Ronquist et al. 2012). The GTR+G model was chosen as the optimal model of nucleotide substitution using the Akaike information criterion (**AIC**; Burnham and Anderson 2002) as implemented in IQ-TREE (Trifinopoulos et al. 2016). The Markov chains were run for 1,000,000 generations and sampled at each 100 generations, with the first 25% discarded as burn-in.

## Taxonomy

### Begonia pseudoedulis

Taxon classificationPlantaeCucurbitalesBegoniaceae

D.K.Tian, X.X.Feng & R.K.Li sp. nov.

33EBF696-7D38-56CA-A1E3-38664EBF7871

urn:lsid:ipni.org:names:77220007-1

**Figure 1.**
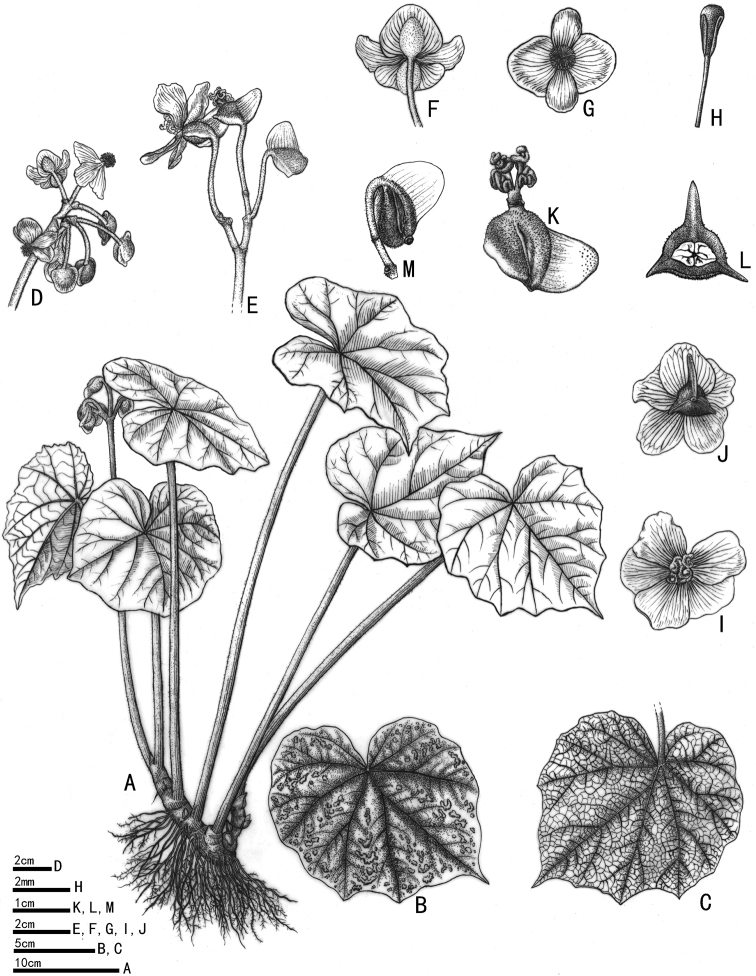
*Begoniapseudoedulis***A** habitat **B** adaxial leaf blade **C** abaxial leaf blade **D, E** inflorescences **F** staminate flower (abaxial) **G** staminate flower (adaxial) **H** stamen **I** pistillate flower (adaxial) **J** pistillate flower (abaxial) **K** ovary and stigma **L** cross section of ovary **M** capsule (Illustration drawn by Yunxiao Liu).

**Figure 2.**
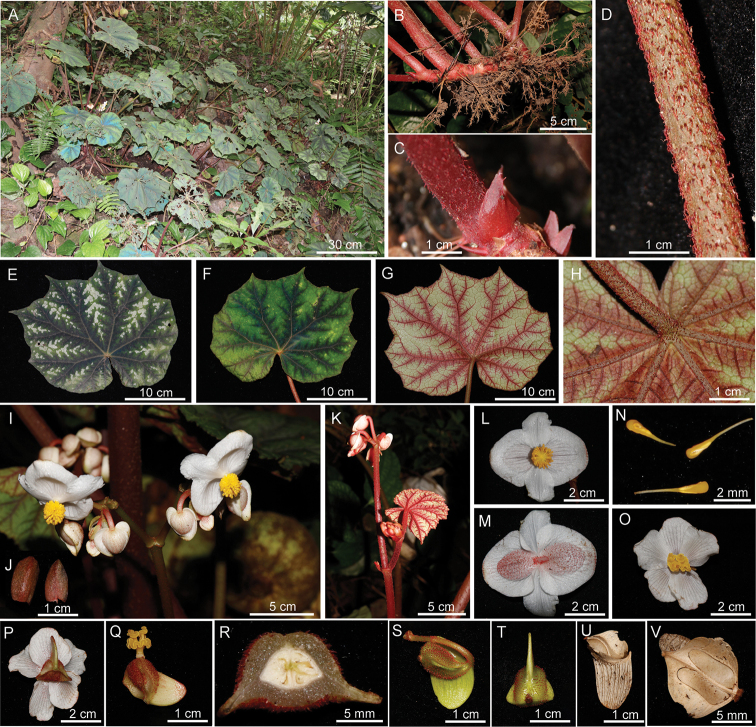
Habitat and morphology of *Begoniapseudoedulis***A** habitat **B** creeping rhizome **C** stipule **D** petiole showing hairs **E** mature leaf blade (adaxial) **F** juvenile leaf blade (adaxial) **G, H** mature leaf blade (abaxial) **I** inflorescence **J** bracts **K** erect stem at anthesis **L** front view of staminate flower **M** back view of staminate flower **N** stamens **O** front view of pistillate flower **P** back view of pistillate flower **Q** ovary with styles and stigmas **R** cross section of ovary **S, T** Immature capsule (different views) **U** dry capsule showing abaxial wing **V** dry capsule showing lateral wings.

**Figure 3.**
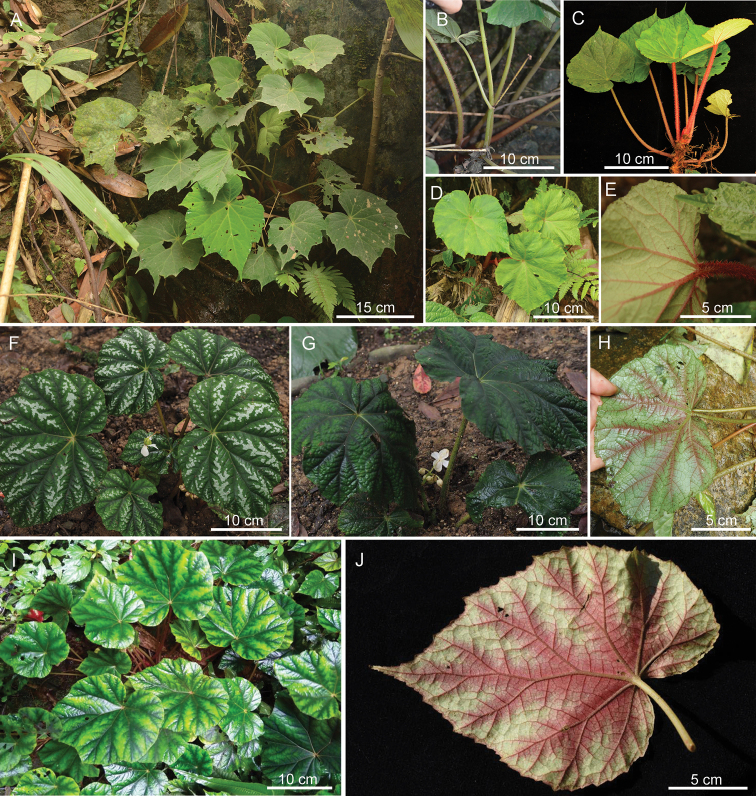
Variation in leaf morphology of different populations of *Begoniapseudoedulis***A–E** the population from Fangcheng, Fangchenggang, Guangxi (**A, B** mature plants **C–E** juvenile individuals showing dense hairs on petioles) **F–H** wuming, Nanning, Guangxi **I, J** daxin, Chongzuo, Guangxi (photos **A–E, J** by Dai-Ke Tian **F–H** by Jun Liu from Zhejiang University; **I** by Chen-Yang Zhao from Daxin County of Guangxi). Note: The population from Daxin, Chongzuo (**I, J**) is only recognized by morphology without molecular evidence.

Figs 1 , 2 , 3 Chinese name: 假食用秋海棠

#### Type.

**Figure 4.**
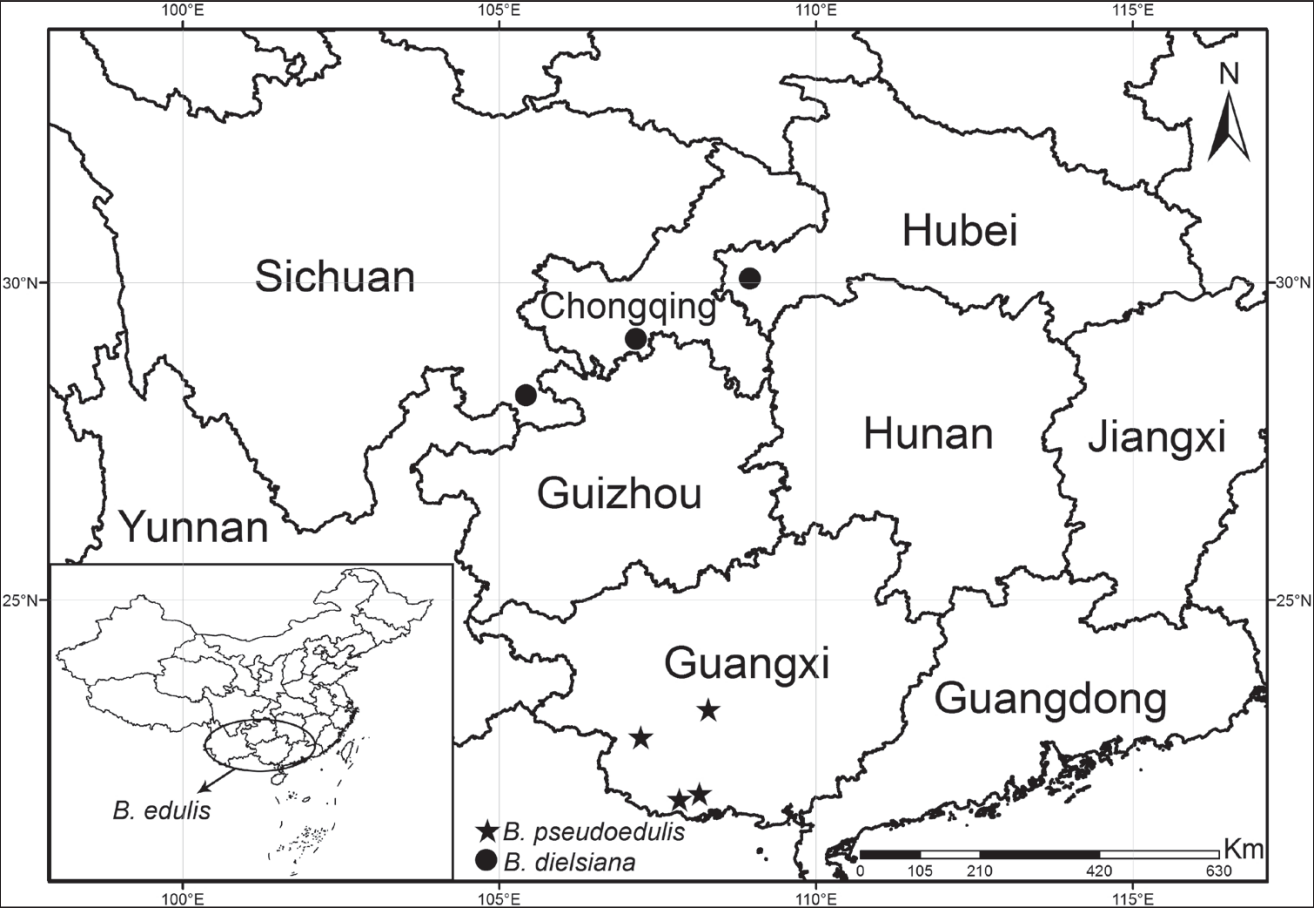
Distribution of *Begoniapseudoedulis* and the allied *B.edulis* and *B.dielsiana*.

**China** Guangxi, Shangsi County (上思县), Shiwanshan (十万山), 21°58'4.71"N, 108°16'50.05"E (Fig. 4), 163 m alt., near a stream under the broad leaf forest, at late period flowering, October 2020, *X. X. Feng*, *Z. X. Liu*, *& R.K. Li*, *FXX201001* (***holotype***: CSH0185896, CSH!; ***isotypes***: CSH! & SYS!).

#### Diagnosis.

The new species shows high resemblance to both *B.edulis* and *B.dielsiana* from the same section (sect. Platycentrum) in their stout and creeping rhizome, erect stem at anthesis, asymmetric ovate and chartaceous leaf blade, palmate venation, dichasial cyme, four tepals of staminate flowers, unequally 3-winged capsules. However, it differs from the latter two mainly by its shorter (vs. taller) plants, variation (variegated more than pure green vs. green or dark-green for *B.edulis* and pure green for *B.dielsiana*) in leaf color, hairy (vs. glabrous or nearly so) petioles and red hairs (vs. glabrous or nearly so) on abaxial surface of outer tepals, stable five (vs. usually five and rarely six for *B.edulis*, and more six than five for *B.dielsiana*) tepals of pistillate flowers.

#### Description.

Perennial evergreen herb, monoecious, 30–50 cm tall. **Rhizome** stout and creeping, ca. 10–12 cm long and 10–15 mm in diameter, internodes obvious and crowded; erect stem only at anthesis, usually reddish-brownish, rarely green, sparsely pilose. **Stipules** membranous, triangular, reddish or green, glabrous. **Leaves** 6–8 basal and 2–3 aerial, petiole pale- to reddish-green, 18–50 cm long, 5–10 mm thick, densely red pilose, 3–5 mm long at young stage and then sparsely short reddish-brownish or gray hairs in mature; leave blade variable in shape and coloration, usually widely ovate, 12–23 × 9–22 cm, adaxially dark-green along main veins or evenly green, rarely white spotted, puberulent, abaxially purple-red along veins or evenly green, subglabrous except main veins, base strongly oblique-cordate, margin triangularly denticulate to shallowly lobed, apex caudate. Venation palmate, primary veins 7–8, adaxially slightly concave, abaxially convex. **Inflorescences** arising from erect stem at anthesis, dichasial cymes branching only once, peduncle 16–23 cm long, red short strigose, flowers unisexual, 5–8 flowers per inflorescence; bracts membranous, oblong triangular to widely ovate, reddish-brownish, 8–15 × 4–8 mm, glabrous. **Staminate flower**: pedicels pink, 2.0–2.5 cm long, hairy nearly same as peduncles; tepals 4, white or nearly so, outer 2 broadly ovate, 18–23 × 17–22 mm, middle part thicker, adaxially concave, abaxially convex, red short strigose, veins distinct, apex obtuse, margin entire; inner 2 nearly obovate, ca. 18 × 14 mm, glabrous; androecium spheroid, ca. 11 mm across; stamens numerous, ca. 4–5 mm long; filaments fused at base, anthers yellow, clavate, base cuneate, ca. 2 mm long. **Pistillate flower**: pedicels pink, 2.0–2.5 cm long, hairy nearly same as peduncles; tepals 5, white or nearly so, irregularly suborbicular, fan-shaped or broadly ovate, sub-equal, 16–19 × 15–17 mm, abaxially sparsely red short strigose, apex obtuse; ovary yellowish-green, trigonous-ellipsoid, 12–14 × 8–9 mm (wings excluded), red hispidulous; 2-loculed, placentation axile, placentae bifid per locule; styles 2, fused at base, yellow, ca. 7–9 mm long; stigma U-shaped, spirally twisted. **Capsules** nodding, trigonous-ellipsoid, ca. 18 × 8–10 mm (wings excluded), yellowish-green, red hispidulous; wings 3, unequal, abaxial wing semicircle-shaped or rectangular, ca. 15 mm long; lateral wings narrow, 3–4 mm long.

#### Additional specimens examined.

**China Guangxi**: Fangchenggang District (防城港区), Dongzhong Town (峒中镇), Nameng Village (那蒙村), roadside of S325, near stream under bamboo forest, 21°38'32.63"N, 107°35'48.91"E, elev. 380 m, 1 April 2015, *Dai-Ke Tian, Li-Zhi Tian, TDK2423* (CSH!); Dongzhong Town (峒中镇), Dakeng Village (大坑村), Maan’ao (马鞍坳), 21°38'25.32"N, 107°39'34.40"E, elev. 650 m; Wuming County (武鸣县), Liangjiang Town (两江镇), Daming Mountain Nature Reserve (大明山保护区), 23°25'38"N, 108°27'35"E, elev. 550 m, *Dai-Ke Tian, Yan Xiao, Yi Tong & Li-Zhi Tian, TDK3008* (CSH!); Wunming County, Liangjiang Town, 23°26'19.74"N, 108°24'31.92"E, *Ya-Hong Gao TDK4001* (collected at Shanghai Chenshan Botanical Garden from introduced plants)

#### Distribution and habitat.

Currently known from four localities with elev. 160–650 m. (Fig. 4). It grows in shaded environment along the stream or near waterfall under the broad-leaved forest.

#### Phenology.

Flowering August–October, fruiting September–November.

#### Etymology.

The specific epithet “*pseudoedulis*” refers to its similarity to *B.edulis*, because both are easily confused based on appearance when the inflorescences and flowers are invisible.

**Conservation status**. Only four populations with under 1000 estimated individuals have been found so far in three counties of Guangxi. Each population consisting of approximately 20–300 individuals is distributed in no more than 200 m^2^ area. The population size is prone to decrease by illegal collection for medicinal and ornamental uses. Considering the disturbance of human activities and narrow distribution, *B.pseudoedulis* is currently assessed as “Near Threatened” (NT) according to the IUCN Red List Categories and Criteria (IUCN, 2019).

##### Molecular Analysis

**Figure 5.**
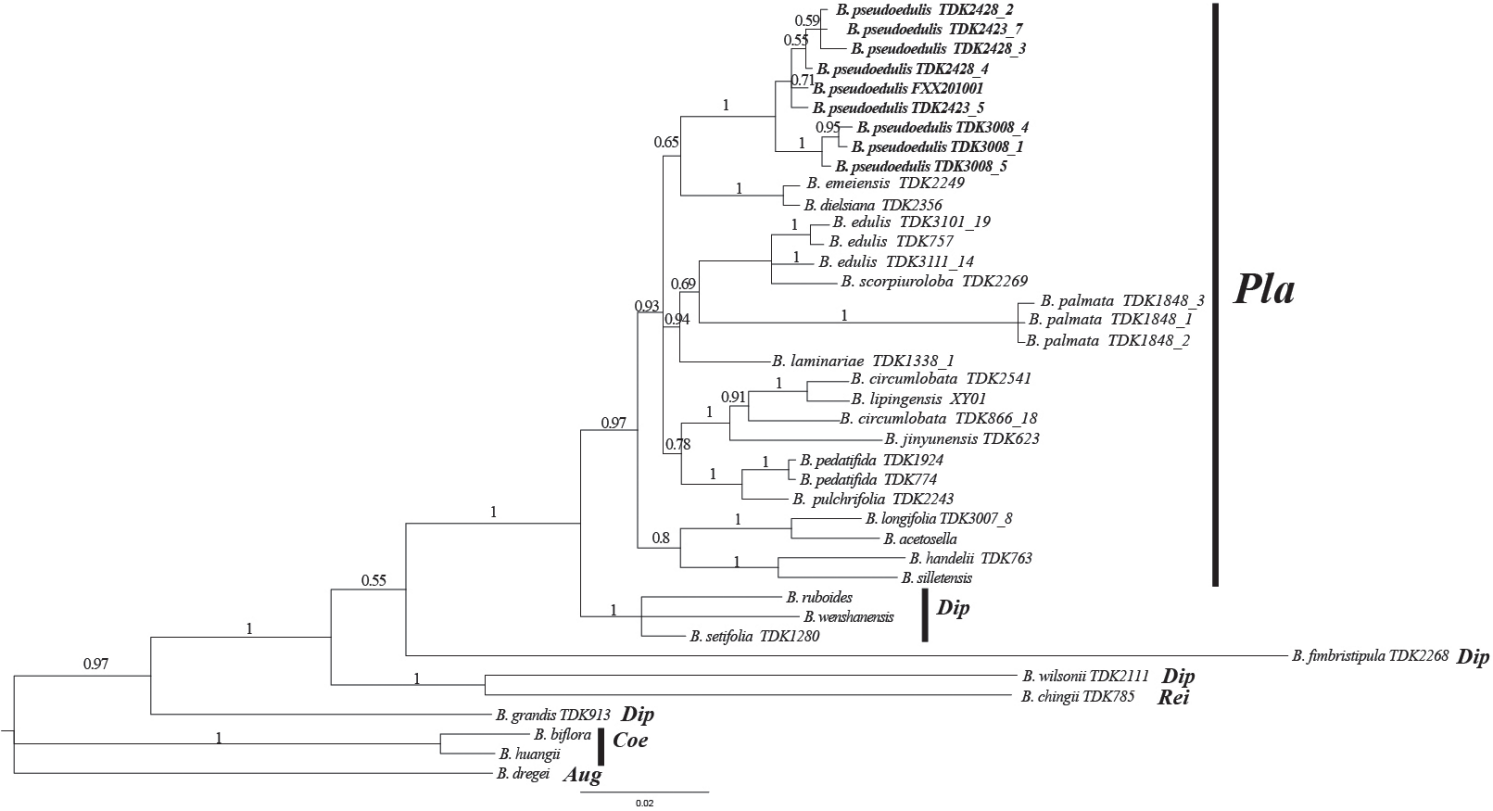
Bayesian inference of the phylogenetic position of the newly described *B.pseudoedulis* within sect. Platycentrum based on nuclear ITS sequences.

The ITS data set containing 40 accessions represented 26 species, four main sections of Begonia in China and one section from Africa (Table 1). The aligned matrix of ITS region was 664 bp. The result of Bayesian inference analysis was shown in Fig. 5. The sect. Platycentrum appeared monophyletic with a high Bayesian posterior probability (bpp = 0.97). The putative new species was a monophyletic lineage (bpp = 1) and sister with *B.dielsiana* and *B.emeiensis*. *Begoniaedulis* with the highest morphologic resemblance of *B.pseudoedulis* formed another clade.

Nodes with bpp < 0.50 have been collapsed. Sectional placement of taxa is indicated by the following abbreviations: *Aug*: *Augustia*, *Coe*: *Coelocentrum*, *Dip*: *Diploclinium*, *Pla*: *Platycentrum*, *Rei*: *Reichenheimia*. The numbers after the species names indicate different populations. The samples of new species are indicated in bold letters.

## Discussion

*Begoniapseudoedulis* is assigned to B.sect.Platycentrum by its 2-loculed ovary, placentation axile, and placentae bifid per locule (Gu et al. 2007). In the same section, it is most similar to *B.edulis* and *B.dielsiana* and *B.emeiensis* in morphology (Fig. 4).

**Figure 6.**
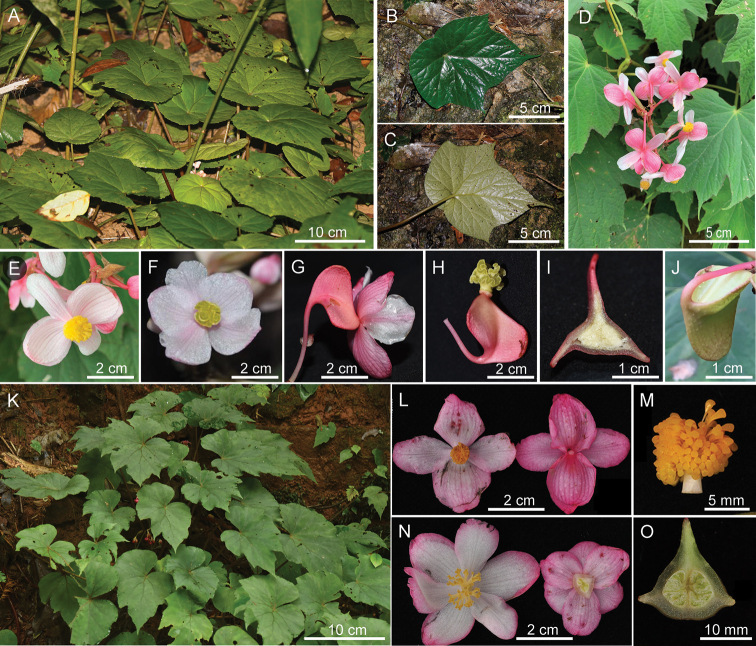
*B.edulis* (**A–J**) and *B.dielsiana* (**K–O**) for comparison to *B.pseudoedulis***A** habitat **B, C** leaf blade (adaxial and abaxial) **D** inflorescence **E** staminate flower **F, G** pistillate flowers **H** ovary & stigma **I** cross-section of ovary **J** immature capsule **K** habitat **L** staminate flowers **M** androecium **N** pistillate flowers **O** cross-section of ovary.

The new species shows high resemblance to *B.edulis* in stout creeping rhizome, erect stem at anthesis, palmate veins, chartaceous leaf blade, dichasial cyme, unequally 3-winged capsule (Table 2, Fig. 6; Gu et al. 2007). However, it can be distinguished from *B.edulis* mainly by its densely red hirsute (gradually wide down to base) (vs. glabrous to subglabrous) petioles and red hispid (vs. glabrous or nearly so) on the abaxial surface of outer tepals of staminate flowers, ovary and fruits, and usually variegated and seldom pure green (vs. evenly green or dark green) leaf blades.

**Table 2.** Morphological comparisons among *B.edulis*, *B.dielsiana* and *B.pseudoedulis*.

Character	*B.edulis*	*B.dielsiana*	*B.pseudoedulis*
Plant height	up to 1.5 m	up to 90 cm	30–50 cm
Petiole	green to red, glabrous to subglabrous	green, glabrous to subglabrous	usually reddish, seldom green, densely hairy at young stage
Leaf color	adaxially green or dark green, abaxially light green or purple red	both sides green	adaxially green but dark green along main veins, abaxially light green and purple red along main veins, seldom evenly green
Inflorescence	peduncles green or red, glabrous to subglabrous	peduncles green, glabrous to subglabrous	peduncles red or occasionally green, densely red hairy particularly at young stage
Staminate flower	pedicels and tepals glabrous	pedicels and tepals glabrous	pedicels and outer tepals red hispidulous
Pistillate flower	pedicels glabrous, tepals 5 rarely 6, glabrous, unequal, inner one smallest	pedicels glabrous, tepals 6 rarely 5, subequal, glabrous	pedicels hairy, tepals 5, subequal, outer three hairy
Ovary and capsule	glabrous	glabrous	Hairy
Flowering time	Jun.–Sep.	Jul.–Aug.	Aug.–Oct.
Distribution	100–1500 m, Hunan, Guangdong, Guangxi, Guizhou, Yunnan; Vietnam	950–1350 m, Chongqing, Hubei, Hunan, Sichuan	160–650 m, Guangxi only

*B.emeiensis* shares almost the same morphologic characters and geographic distribution (Central China) with *B.dielsiana* and could be treated as a synonym or variety of *B.dielsiana* based on the unpublished data from Daike Tian’s lab. They can be easily distinguished from the new species in having evenly green leaves with glabrous to subglabrous petioles, glabrous flower tepals, and usually six (less in five) tepals of the pistillate flowers (Table 2, Fig. 6).

Among these three species, *B.edulis* is most commonly seen in China usually with large populations, and has the widest distribution followed by *B.dielsiana.* The new species has the narrowest distribution range, which is only found in Guangxi. Although the three species share high similarity in morphology, the molecular evidence strongly supported that they are three different taxa.

## Supplementary Material

XML Treatment for Begonia pseudoedulis
